# Household Air Pollution Intervention Implications: Findings from Qualitative Studies and a Field Trial of Clean Cookstoves in Two Rural Villages in India

**DOI:** 10.3390/ijerph13090893

**Published:** 2016-09-09

**Authors:** Ashraful Alam, Nanda Tawale, Archana Patel, Michael J. Dibley, Sunil Jadhao, Camille Raynes-Greenow

**Affiliations:** 1 Sydney School of Public Health, University of Sydney, Sydney 2006, Australia; michael.dibley@sydney.edu.au (M.J.D.); camille.raynes-greenow@sydney.edu.au (C.R.-G.); 2 Lata Medical Research Foundation, Nagpur 440022, Maharashtra, India; meetbhairavi@hotmail.com (N.T.); dr_apatel@yahoo.com (A.P.); mphsunil@gmail.com (S.J.)

**Keywords:** air pollution, focus groups, cooking, family characteristics, Asia, India

## Abstract

Exposure to household air pollution is estimated to be the 3rd largest contributor to the global burden of disease and the largest contributor in South Asia. Unacceptability of improved cook stoves by the intended user has been identified as a crucial factor hindering uptake and sustained use. We conducted a qualitative study to understand the socio-cultural factors that influence acceptance of improved cookstoves and conducted a systematic field trial in two rural villages in Maharashtra, India. The qualitative study used semi-structured in-depth interviews and focus group discussions. We included women primarily responsible for household cooking, their husbands, senior women in their households, and community health workers. We also conducted kitchen observations. The results indicated low awareness and knowledge of the health risks associated with traditional cookstove use although high prevalence of household air pollution (HAP) exposure symptoms among all groups. Women were resigned to using traditional cookstoves although they did not like them. The field trial findings were dominated by responses concerned with convenience and health advantages. We identify important issues to be considered when introducing an improved cookstove programme that will increase acceptability and potentially sustained used of improved cookstoves.

## 1. Introduction

The 2010 Global Burden of Disease study identified exposure to household air pollution (HAP) from solid fuels as the third largest contributor to the global burden of disease and the largest contributor in South Asia [1,2]. Incomplete combustion of biomass fuels using traditional cookstoves in low and middle-resource settings is a primary cause of HAP [3]. Traditional cookstoves and solid fuels are almost universal in low resource settings and nearly 2.4 billion people worldwide and 75% of South Asian use solid fuels as their main source of domestic energy for cooking and heating [4]. Women and young children are the most exposed to HAP, as they spend the greatest amount of time inside the home and in close proximity to a stove often in confined spaces with poor ventilation [5]. There are many indirect risks associated with HAP exposure and traditional cooking methods that extend beyond health, into livelihood, and productivity further exasperating poverty [4]. Biomass combustion also increases environmental degradation by contributing to global warming [6,7], and deforestation [8].

One potential intervention to reduce exposure to HAP is through low-emission or ‘improved’ cookstoves. Improved stoves exhibit a 20%–50% reduction in exposure to particulate matter and carbon monoxide compared to traditional stoves in India [9,10] and Mexico [11] and have demonstrated reduced health risks [12,13]. Nevertheless, sustained adoption of cleaner cookstoves remains very low [5,14].

The reasons for low adoption of cleaner cookstoves are multifactorial, and are attributable to both end user issues such as motivation and cost and also distribution and product failures by not meeting user needs [15,16]. Successful implementation of any cooking intervention to address HAP requires substantial understanding of the social and cultural determinants that influence cooking behaviours and end-users preferences and needs of cookstoves. This study aims to address this gap by describing attitudes, perceptions, and knowledge of HAP, investigating current cooking practices, observing kitchens and conducting a systematic user experience field test of improved cookstoves, among two rural communities in India.

## 2. Materials and Methods

### 2.1. Study Location

The study was conducted in two rural villages in Maharashtra, India, within approximately 70 km of Nagpur city. The region has three main seasons, a very hot summer with temperatures regularly reaching 40 °C Celsius between March and July, followed by a monsoon. Winter is mild with mean daily temperatures of ~20 °C.

### 2.2. Participants

Participants included (i) women who self-identified as being the primary family cook in the household; (ii) their husbands; (iii) senior women in their household; (iv) ‘Anganwadi Workers’ [17]; (v) Accredited Social Health Activists (ASHA), community based health volunteer [18]; and (vi) Auxiliary Nurse Midwives (ANM) in the study villages. We generically refer to the last three groups as community health workers (CHWs) throughout the paper.

The research team in India contacted the CHWs of the local primary health center who was assigned to the village. They were asked to suggest potential families to approach to participate in the study. The research team explained the selection criteria of respondents and were asked to recommend families from various socioeconomic strata (e.g., caste, and higher and lower socioeconomic status). The CHWs who had a good knowledge of the community nominated households that were typical of the community. The field research team then approached the senior person in the family and sought permission to talk to the women in the household. The women to be interviewed in the household were finally selected based on their self-identification as the main cook.

### 2.3. Study Design

We conducted a mixed methods study, with two main components (Table 1). The first component included the qualitative methods; in-depth interviews with primary cooks, senior women and the CHWs, and focus group discussions with husbands, and a structured kitchen observations in all participating households. The second component was a systematic field-test of clean cookstoves in the village households.

The local research staff (Sunil Jadhao and Nanda Tawale) recruited and enrolled all participants (with assistance from the auxiliary nurse midwife in each village) and collected all data. Local research staff were given specific training for this project by Ashraful Alam, an experienced medical anthropologist and Camille Raynes-Greenow, a senior epidemiologist with social research methods expertise. The study was overseen by Alam, Raynes-Greenow and Archana Patel through weekly meetings that Alam attended by visiting Maharashtra and online. The interviews and focus groups were conducted in the local language and were guided by a flexible qualitative data collection protocol that was developed by Alam and Raynes-Greenow together with the team. The protocols approximated each other regardless of the data collection method, as the aims were similar. The protocols were pre-tested with participants from the same villages who were subsequently excluded from participating. Interviews were conducted in the household of the participant, and Focus Group Discussions (FGD) in the *Gram Panchayat* office in each village.

### 2.4. Field Test of Improved Stoves

We identified five improved cookstoves models that were locally available in Nagpur. The stove models were: (1) Envirofit B-1200 WOOD (Black) (manufactured in China by the Envirofit International, Inc., Fort Collins, CO, USA); (2) Envirofit G-3300 WOOD (Red) (Envirofit); (3) Greenway Grameen (manufactured by Greenway Grameen Infra, India); (4) Onil Plancha (HELPS International, Addison, TX, USA); and (5) Purti pellet stove (produced locally by the Purti Alternative Fuels Private Limited, Maharashtra, India). Each model (and fuel if necessary) was provided to each participating household for one week, and then rotated. The field researchers counselled the users of the stove on the advantages of the improved stoves, and potential challenges the users might encounter in using them as well as how to address the challenges. Feedback from the qualitative research findings were used to develop the content of the counselling. Each household also received written and verbal training regarding installation, proper use, safety and demonstrations, possible hazards of the stove model and how to manage these. A pictorial leaflet with illustrations for troubleshooting was attached to a kitchen wall, and field staff were also available by mobile phone to assist with troubleshooting and managing any technical problem. All technical advice and stove demonstrations were repeated for each new installation of each model for each household. At the end of each test week an interview was conducted with the woman identified as the primary cook. Each household also received verbal communication regarding the risk of HAP exposure.

### 2.5. Data Analysis

All data were audio-recorded then transcribed verbatim into Marathi (the local language) and then translated into English. A sample of the translations were discussed (by Alam and Raynes-Greenow) for quality. We applied an inductive coding procedure where themes were derived empirically induced from the data that were related to our research questions [19]. Initially two transcripts were independently coded for emerging themes by four authors (Jadhao, Tawale, Alam and Raynes-Greenow), who then together discussed and developed the final codes. All transcripts were manually coded (Jadhao and Tawale) according to the final thematic codes, and then independently reviewed (Alam) to ensure inter-coder reliability. We applied an inductive thematic approach [20] for analysis. The analysis team then subsequently discussed the text pertaining to each thematic code, after several discussions these were consolidated and summarised in a document for each theme with relevant quotes and text tables. Themes were triangulated using data collected through various methods and from multiple groups of respondents.

### 2.6. Ethics

All study participants gave their informed consent for inclusion before they participated in the study. The study was conducted in accordance with the Declaration of Helsinki, and the protocol was approved by the Ethics Committee of the Lata Medical Research Foundation, Nagpur, Maharashtra, India that is registered with the United States Office for Human Research Protection (approval reference number: RPC10).

## 3. Results

### 3.1. Participant Characterises

There were 14 women who agreed to participate in the interviews and stove field testing and self-identified as the primary family cook, 13 husbands of these women (one husband was unavailable), and 12 senior women, all mothers-in-law of the primary cook (two were unavailable). Additionally, we interviewed four CHWs (all female, two ‘Anganwadi Workers’, one ASHA, and one ANM. There were no refusals to participate. All households reported farming, as their primary income. Women who identified as the primary cook were aged between 20 and 25 years and were multiparous. Most (primary cook) women had some formal education, between 3 and 10 years, and their main occupation was domestic responsibilities, although most also reported farming duties. Most households self-identified as being in a low socio-economic bracket, although some were more affluent. Multi-generational and multiple families sharing one home was also typical and the mean family size was seven. Families belonged to a variety of Hindu castes. Both villages were connected to Nagpur city via paved roads.

### 3.2. Current Stove and Fuel Practices

All participants reported using a traditional clay (or sometimes called mud) stove as their primary stove, although many homes also had a liquid petroleum gas (LPG) stove and several had a kerosene stove. Most families had two traditional clay stoves, one inside the house for cooking meals and another outside the house for heating water. These were called ‘chula, chulha or chul’ interchangeably in these villages. Families who were relatively more affluent used both a traditional stove and an LPG stove. LPG stove use was constrained due to fuel cost. Subsequently, when LPG stoves were used it was for preparing snacks (that required little fuel) or for cooking meals quickly for hospitality for unplanned guests or for similar occasions when something needed to be heated quickly. Kerosene stoves were used by poorer families in the same way as the more affluent families used the LPG stoves.

“In the morning time I use LPG and in the evening time mud stove. Because morning time has very much busy schedule and my husband has to go for work, for that I need to cook fast.”

A woman

### 3.3. Usual Cooking and Stove Practices

The daily meals included two main meals, in the morning before leaving for farming work and another in the evening. A third lighter meal (e.g., lunch) was not a common practice. However, tea using boiled milk was made at least twice a day, and more milk would be warmed for households with small children. The average amount of time spent preparing and cooking a meal was between one to two hours per meal depending on the size of the family. If visitors arrived a lighter meal would be cooked.

Before stove use commenced, small twigs and branches were fed into the front feeder of the stove, and ignited. Crop residue (Tur twigs), or cow dung cakes were also used for ignition with a match and set into the prepared stove, when the fuel was damp a piece of paper dipped in kerosene, was used. Cow dung was a complementary fuel and was reported to burn faster (i.e., be less efficient) and produce substantially more smoke compared to firewood. As the cooking process came to an end, the fire self-extinguished. Water, mud or ash were sometimes used to extinguish the fire.

The daily food preparation began with tea making followed by boiling water to cook the rice and dal. In the meantime, vegetables and spices were prepared. Once the dal and rice were cooked, the vegetable curry was cooked. Meanwhile dough for chapattis or rotis (the words used interchangeably in these villages) was prepared, and cooked once the vegetable curry was finished. While cooking women sat or squatted on the ground with the cookstove at a height of ~45 cm, which allowed for easy stirring and handling of pots. Three burner clay stoves were the most preferred ones.

*“In the morning I prepare tea, boil water for all five members, then meal like dal, chapatti, rice and vegetables. This cooking finishes at 11* [a.m.]. *Then the chula *[stove]* again lits in the evening at 5 for preparing tea, dal, chapatti, rice and vegetables. Sometimes for tea, I lit chula in the afternoon. We have only one chula that we use for all purposes.”*

A woman

Stoves were also used in the morning for heating water for bathing. This was done in a large pot on the outside stove. This was a seasonal activity restricted to the cooler months.

There were other modifications to cooking practices according to the season, and the number of people eating. Changes included different stove uses and types, and the cooking location. Indoor cooking was the preferred place for cooking during the monsoon. Outside courtyard cooking was the place for cooking large quantities of food (such as a marriage or blessing ceremony). This was done using a tripod brick stove.

*“When a large number of relatives comes to house we build separate large cook stove of bricks outside of the house for cooking. Also when we prepare papad, dhapode* [in large quantities] *similarly we build large cookstove of bricks outside.”*

A woman

“There is variation throughout week and year. I use mostly the clay cook stove throughout the year, but in four months of summer, I prefer the LPG stove. In remaining eight months we use the clay stove mostly.”

A woman

### 3.4. Fuel Collection, Cost and Storage

The type of fuel used depended on availability in the local area. In this region with orange tree orchards, fuel from these trees was the dominant fuel type although not exclusive. Dried cow dung, flattened into cakes and crop residue were also used. Fuel was collected from their own farm trees, or they paid neighbours to collect from their farms. Families also reported collecting fuel from the nearby jungle and from the road side.

*“Some common names *[of trees]* we know like babul, neem, parathi and other crop residue, there are many more but we don’t know the names. Wherever we found nearby jungles and farm we collect it and bring it* [to burn in the stove].*”*

A mother-in-law

Fuel collection was both purposive and opportunistic. Women reported spending between one and two hours a day collecting firewood, but it was regarded as a secondary activity to regular activities and perceived as an opportunity for socialising. Men also participated in firewood collection and this was also mostly opportunistic while conducting their daily activities. Crop residue (e.g., husks) was also used which was mixed with cow dung to make cakes. Most people collected fuel wood for free, from their own farms. Some households reported paying labourers to cut and collect firewood on their lands. The minimum labour cost was reported at ~Rs. 200 per day, or ~Rs. 600–800 per year for an average household. Some households also purchased old trees that were being replaced in the orchards at ~Rs. 25–40 each.

Fuel storage was also a consideration for households, and this included keeping the firewood dry and also protected from theft. Storage was usually in the front or backyard, and only a small amount of firewood sufficient for immediate use stored in a room near the stove. During the monsoon, the firewood was moved undercover if possible, and in some cases it was inside the house.

### 3.5. Perceptions and Satisfaction of Traditional Stove Use

Traditional stoves were ubiquitous and we asked participants their reasons for using traditional stoves. Most were surprised with the question as they did not consider they had a choice and reported ‘tradition’ as their main reason for continued use. The cost was also an important reason. Participants described how traditional stoves were handmade using locally available material with little or no cost, virtually no maintenance costs, and fuel was generally freely available.

*“No option…Yes, the advantage of this chulha* [stove] *is that as we are poor so is good for us. Fuel is free and easily available here; also we can’t buy LPG stove or LPG cylinder.”*

A woman

Overall most women were dissatisfied with traditional stoves, and described many disadvantages. These were categorised into two main sub-groups, (1) inconvenience; and (2) health annoyances. Inconvenience issues included pots being charred which subsequently required substantial extra cleaning, black soot staining the walls, the room becoming hot and smoky, problems and delays with stove ignition, and large amounts of fuel required. The season was also an additional factor related to inconvenience. The monsoonal rains caused greater moisture content in the fuel resulting in slower ignition and increased smoke, and the high summer temperatures made indoor cooking very uncomfortable.

The health annoyances included eye redness and irritation, coughing, allergy type symptoms, chest pain, feeling dizzy when cooking, and accidental burns for both women and children.

*“The smoke produced by it* [traditional stove] *causes irritation of my eyes. My eyes become red due to smoke, heat generated by it troubles me. While cooking many time the flames cause burn to the body, blackness of pots; … *[traditional stove]* more firewood is required.”*

A woman

*“We are not happy with the use of the current cookstove. In rainy season more smoke is generated and in summer due to heat temperature rises so we have trouble in both seasons. … My wife’s eyes burn from the smoke generated *[by the clay stove]*. It blackens the pots, burns hands and burns cloths. Mainly we don’t have option so we are using the current clay stoves.”*

A husband

Having no other options and lack of financial ability emerged as the dominant reasons of continuing using traditional stoves in many interviews. For example, a women mentioned:

*“Look, our financial condition is not good, so we don’t have much options. Right from the beginning I am using the clay cook stove; in my maternal home also we had that clay stove. We don’t have options, so we use that. Also, we have abundant firewood, cow dung,* [and] *agricultural waste which can easily be used as fuel, so we are using them. Even though we have LPG, we still are using the clay stove. *[Because of all these factors]* we are much comfortable with our clay stove.”*

A woman

Overall there was agreement on the disadvantages of traditional stoves between men and women. Mothers-in-law were the exception, and contrary to the rest of their family no mother-in-law reported any disadvantage of using traditional stoves.

“I didn’t find any disadvantages of my clay stove throughout my life. The women from my era were habitual to use that type of chulha. These new generation girls have the problem with clay stoves.”

A mother-in-law

### 3.6. Awareness of Household Air Pollution

There was some awareness of HAP exposure and this was described through their experience of symptoms such as eye irritation, and coughing, rather than knowledge of HAP risks.

*“Chest pain, dizziness, cough, eyes gets sore and pain. These are very common problems and it happens in day-to-day life but we don’t know much about its related health problems. We never realized that problems in our lifetime *[related to HAP exposure]. *Yes there may be some effect of that on health of them,* [such as] *burning of eyes, coughing, etc.”*

A husband

Mothers-in-law were the exception who despite not reporting any disadvantages of traditional stoves were more likely to attribute symptoms to the HAP exposure.

“Whenever I prepare food for a large number of people my eyes get sore and irritated think it may be. Yes eyes get sore, watery, inflammation of eyes, sometimes feels dizziness, cough, etc.”

A mother-in-law

Some respondents perceived that an exposure to HAP could harm the health of children “because they have less power to tolerate the smoke”, but did not mention any specific impact on themselves. Overall women and their family members were more aware of the impact of HAP exposure on children’s health than that on the health of their own.

The health workers all were aware of risks associated to HAP exposure but were very vague.

“It may be because whenever mother breathes, it directly affects the baby in the womb. We can’t say exactly what happens but possibilities are there. No, we don’t know much about that. We never realized that in our lifetime. … Yes, there may be some effect of that on health of them. Burning of eyes, coughing, etc.”

—An ASHA

Regardless of the participants’ experience of these symptoms most reported that they did not usually cease work due to symptoms.

### 3.7. Influential Persons

We intended to know about the key persons who the women should seek advice from to make a decision about replacing the traditional stove with an improved stove. The *Sarpanch* (elected Head of the Gram Panchayat the local self-government organisation at the village or small town level) and the members of the Panchayat were most frequently mentioned by all groups of respondents. Additionally, local health workers indicated women’s mothers-in-law and husbands as the key persons. For younger women the decision about stove choice depended entirely on her family members, as was told by a young woman.

“I am here in this village since one year, so I don’t have much knowledge about the people in village. My decision depends upon my family member, no one else.”

A woman

Women also considered listening to the suggestions from ASHA and Angawadi Workers’, who they regarded as knowledgeable and trustworthy.

Table 2 presents a summary of the results together with key programmatic implications.

### 3.8. Observation of the Kitchen Environment and Location

Data from the kitchen observations supported the interview data. We observed changes to the placement of the stoves related to seasons; in the dry season, courtyards were often used for cooking as it was more spacious, and provided more natural light and fresh air than the indoor kitchen. However, in the rainy season, almost all of the cooking was moved to closed spaces. Affluence was an important factor that modified kitchen placement. More affluent households had a separate kitchen but poorer families used a single multi-purpose room. Ventilation was also modified by affluence, with more affluent households having windows and or doors. We observed a striking visual difference between ventilated rooms and non-ventilated rooms used for cooking as in the later, walls and ceiling were covered in black soot. Most kitchens of affluent families were made of bricks and cement concrete whereas poorer households were made of mud walls. We did not observe any chimneys.

### 3.9. Field Test of Stoves

Women from each household reported their experience of the stove field test. Convenience, difficulties and suggested modifications of the stoves relative to all test stoves and their usual traditional cookstoves were considered. The primary finding from these evaluations was the perceived increased usefulness and greater convenience of the improved stoves relative to traditional stoves. Above all other factors, the taste of the cooked food was considered as a qualifier of how the stove was rated. Identified issues included, reduced smoke, fuel consumption, reduced pot and utensil charring, and reduced cooking time were all important.

Time saving stoves such as those with two burners were considered more efficient and hence were favoured over single burner stoves. Fast ignition was also an important time saving requirement. Stoves that needed less supervision were considered more time saving as they allowed the women to attend to other chores while cooking continued. Cost and anticipated costs were also considered as convenience factors, such as a pellet stove was considered less convenient as ongoing purchase of pellets was an anticipated financial burden. Fuel issues although not related directly to cost were also discussed. Some of the stove designs had narrower feeder mouths and these required straight, or smaller twigs which impacted on their fuel collection practices, and fuel feeding. Another stove that required kerosene for ignition and wood for burning was unfavourably rated. Smoke emission was also a major factor, with women noticing the difference between the models and having a strong preference for reduced emissions. Taste was the final deciding factor, as food had to taste similarly to food cooked on traditional stove. Women considered all issues including convenience, difficulties and suggested modifications, and then rated them (Table 3).

## 4. Discussion

There have been more than 30 years of both government and non-government funded programs to distribute clean stoves around the world in resource poor settings. Many of these have been framed within a developmental context, and have mostly been unsuccessful in terms of end-user adoption and long-term sustainability of stove use. Most programs have been developed with little consideration of the end-users needs and have not been informed by thorough knowledge of current cooking behaviours, preferences and/or end-users experience of the stoves. This study thoroughly describes current cooking practices, and preferences, triangulated with kitchen observations and a systematic household field test of clean stoves. These results provide a set of programmatic implications to consider and review before implementing a new stove programme whether it be for a development, health or an environmental programme.

We found that there was low knowledge of the risks associated with HAP, however most households had experienced symptoms related to HAP exposure. Experience of symptoms and knowledge of risks are different albeit intertwined issues. Neither experience of symptoms nor knowledge are sufficient to modify behaviour although linking adverse health effects with symptom experience has been suggested as an useful tool to change behaviour [21]. Further, a reduction in adverse health effects with improved stove use was identified as an enabler for adoption [22]. In our study, participants were concerned with their children’s exposure. Incorporating child risk information into an education programme may increase ICS adoption. An obvious avenue for such an education programme is through CHWs, who are already disseminating health information. CHW’s positive role in effective delivery, acceptance and adherence to an intervention is well documented provided they have management support, focused tasks and proper training [23,24,25].

All households reported that cost and or anticipated cost was an important factor related to current use or stove preferences in the field testing. A review of factors associated with improved stove uptake reported both fuel and time ‘costs’ were important incentives, and higher socio-economic status was an enabler to adoption [22]. In our study cost was considered more broadly. (1) Poorer families had more restricted LPG use, and tended to use kerosene in the same way that more affluent families used LPG due to the cost; (2) all women reported the ‘cost’ of their time of cleaning charred pots; (3) women rated the stoves in the field test lower if they needed to purchase fuel, or collect a specific shaped twig; and (4) women rated the stoves more favourably if they did not need constant supervision while cooking, and had two-burners, thus saving time. Financial constraints is a known barrier to adoption of risk-reducing technologies in several low-resources settings [26]. Cash incentives have proven effective for increasing demand for preventive health care [27,28] and adopting new technologies [29]. Providing cash incentives for exclusive use of ICS may be a useful strategy for adoption at least for an interim period until use is normalized, and has recently been suggested [30].

Overall the ICS’s were viewed favourably, which is in contrast to most cookstove intervention studies [5]. This may be a reflection of the short time frame of the ICS stove test, the volunteer nature of the participants or a result of the Hawthorne Effect. Importantly different members of the household had different perceptions. This is an important consideration for future HAP programmes. Other research has identified the importance of including men in the decision making process to purchase an ICS because they are responsible for household finances. Our study highlights the need to involve local community leaders as trusted opinion leaders. We also found that mothers-in-law had different views to their sons and daughters-in-laws and thus it will be important to include all household members in an intervention of ICS promotion.

Most households reported cooking on a combination of traditional and either kerosene or LPG stoves, this practice is known as ‘stove stacking’ [31]. This appeared to be based on cost both fuel and time, and or convenience. Households recognised the benefits of the LPG stoves (faster, more convenient cooking), similar to another study in India, but balanced use with financial cost [32]. Most ICS programs offer a single stove (some a double burner), shifting end-users away from stove stacking will only be sustained if new stoves meet all of the requirements of the household, and this needs to be considered by ICS programmes [31].

The taste of food was the qualifying factor for the field tested stoves. The food cooked using traditional stoves was the ‘gold standard’ and any ICS cooked food had to favourably compare, regardless of any of other factors. Taste was also considered by the participants in another study but it was not the most important criteria, and women in that study said it was likely to be older men who complain about the taste of food cooked on ICSs, again highlighting the differences in household members and the need to have a community approach to stove programmes [33]. Further, participants had suggested modifications for all ICSs, which need to be considered by stove developers, failure to meet end users’ needs has been identified as a barrier to adoption [34].

Although the populations, and conditions (e.g., cooking patterns, weather and fuel availability) may differ, the nature of our findings is generalisable and provides important insights for future work. Our data collection reached saturation, and triangulation of the data from the kitchen observations, different household members and field testing of the stoves ensure reliability and validity of the data. The mixed methods approach and inclusion of the field testing of ICS’s all contribute to the strengths of our study. A further strength of the field testing was that it included household experience with multiple stoves unlike studies where users were limited to a single model [32], or reported opinions of hypothetical scenarios [33]. Feedback from experience with different models will be useful for stove developers.

## 5. Conclusions

Our findings highlight the embedded nature of cooking behaviours and practices, and that adopting a new stove will have implications for all aspects of everyday life which need to be considered. Food preparation and cooking are intertwined with working practices, culture and gender roles, and any intervention that seeks to modify this needs to be developed and implemented based on a thorough understanding of the implications, and any ICS needs to be tested in the field by the intended users. These findings provide key information essential for ICS adoption programs and intervention research, and is aligned with the findings of the recent WHO report that highlights an inter-sectoral approach to improve the environmental determinants of health [2].

## Figures and Tables

**Table 1:** Data collection methods, participants and purpose.

Methods	Participants	Purpose
Semi-structured in-depth interview	Women who self-identified as the household primary cook	Experience of cooking with traditional stoves
		Perceptions of household air pollution (HAP)
		Experience of improved cookstoves cooking
	Mothers-in-law or mothers of the women participants	Experience of cooking with the traditional stoves
		Perceptions of HAP, and perceptions and Experience of cleaner stoves
		Perspective of an influential family member
Focus Group Discussion	Husbands of women participants	Perceptions of HAP, and perceptions and Experience of cleaner stoves by husbands
Key informant interview	Service providers (generically referred to as community health workers) Auxiliary Nurse Midwife attached to the local Primary Health Centre, Accredited Social Health Activist (ASHA), and ‘Anganwadi Worker’	This method generated data on existing cooking practices from local health worker’s perspectives. Information on logistical aspects of possible cleaner cookstove intervention in rural Indian context, and challenges and facilitators of implementing similar interventions in the similar settings
Structured observation	Households of the women participants	At the end of the interview with the woman, the researcher sought for permission to observe the kitchen of the household. The researcher then noted the kitchen characteristics based on a structured checklist (stove type, placement, fuel, ventilation etc.)
Field test of improved cookstoves	Primary cooks and their households trialled the improved cookstove	Experience of cooking with improved cookstove (ICS) to provide information on their experience and identify any likes and dislikes, and preferences

**Table 2:** Summary of all results, including issues, current practice, constraints/barriers, favourable factors and opportunities for intervention.

Issues	Current Practice	Constraints/Barriers	Favourable Factors	Opportunities for Intervention
Knowledge of HAP risks	No education programme in this region	Limited or no knowledge of known risks associated with HAP exposure	Some understanding of exposure and symptoms experience	Training community health workers of risks associated with HAP exposure to improve knowledge
				Opportunities to improve knowledge of the HAP exposure risk, both at individual and community level
		Gender, and generational differences in understanding	Already established mechanism for community education	Opportunities to reduce emissions and thus reduce symptoms experience—thus improving understanding
		General acceptance of HAP symptoms as ‘part of life’		Opportunities to target people who have more knowledge and they become opinion leaders in the village
Experience of HAP exposure symptoms	Limited or no action to reduce HAP exposure	Limited awareness of symptom experience and HAP exposure	Awareness of HAP symptoms	Opportunities for health education
	Some ventilation	Gender, and generational differences in experience of HAP symptoms	Concern for child health	Linking experience of symptoms and risk
		Acceptance of HAP symptoms as part of life		
Fuel, collection storage	Opportunistic collection	Minimal financial cost to household	Considered a burden	Promote awareness of
		Time cost	(time, money other considerations) i.e., improvements are likely to be considered favourably	
	Storage issues, protection from weather, theft	Burden of fuel having sufficient fuel, and correct type, and correct size		Reduced time for fuel collection
	Household expenditure	Some earning from fuel collection		
		Some financial cost to household		Reduced storage requirements
		Sourcing fuel		
	Commodity	Fuel collection activity		Reduced fuel cost
		Seasonal issues (Keeping dry)		
		Security issues—theft prevention		
Kitchen environment and location	Multiple stove use	Seasonal variations	Opportunities for ventilation	A user preferred stove will be easily adopted into usual cooking place
	Multiple burner use	Space	Mobile stoves would be easily adopted into current kitchens	
		Height of stoves		
	Season and event impact kitchen placement	Socioeconomic status impacts kitchen placement, stove/fuel use	Less charring of household walls	
		Cookstoves need to be mobile		
Costs	Cost was embedded in most aspects, cost of time, & cost of fuel	Household budget constraints	Convenience and time saving considered favourably	Highlight any cost saving
	A cognizant factor	Opportunities to highlight cost saving in other areas (time, convenience, reduced fuel costs)		Time and fuel

**Table 3:** Convenience, difficulties and suggested modifications from the improved cookstove (ICS) field test, by stove type, in rank order (top is highest rank).

Stove Model	Convenience	Difficulties	Modifications
Envirofit B-1200 wood (black)	Overall good	Blackens (chars) pots to some extent	Make it a two-burner
	Food tastes good	Requires smaller firewood	Make the mouth broader
	Requires less fuel		
	Produces less smoke		
	Appropriate height		
	Very easy maintenance		
	Safe to use		
Envirofit G-3300 Wood (red)	Overall good	Little too high	Make it a two-burner
	Requires less fuel	Narrower mouth unable to fit usual firewood	Increase broadness of mouth
	Produces less smoke	Takes longer time to get cool	Reduce the height
	Good taste		
	Portable		
	Easy maintenance		
	Safe to use		
Onil Plancha stove	Overall good	Big size needing more space	Increase mouth size to get enough air to ignite quickly
	Food tastes good	Takes more time to ignite	
	Requires fuel less	Needs straight firewood and small twigs	
	Produces smoke less		Reduce the overall size
	Does not blacken utensils		
	Height okay		
	Easy maintenance		
	Two burner save time		
	Safe to use		
	Suitable for pressure cooker		
Purti pellet	Overall good	Requires special fuel (pallets) unavailable in village	Increase the battery life
	Food tastes good	Costs additional to buy pallet	Increase mouth size
	Requires less fuel	Requires daily battery recharge	
	Produces less smoke	Requires kerosene to ignite	
	Height okay	Suitable only for small households	
	Easy maintenance		
	Less blackened utensils		
	Safe to use		
Greenway Grameen	Foods taste good	More time consuming	Make it a two-burner
	Easy maintenance	Requires intense attention	Reduce the height
	Suitable for pressure cooker	Needs more fuel than other improved stoves	
	Safe to use	Need small size firewood	
		Suitable only for nuclear families	
		Overall not a good stove	
